# Remote sensing evidence for population growth of isolated indigenous societies in Amazonia

**DOI:** 10.1038/s41598-023-50127-y

**Published:** 2023-12-17

**Authors:** Robert S. Walker, Mark V. Flinn, Sean P. Prall, Marcus J. Hamilton

**Affiliations:** 1 Department of Anthropology, University of Missouri, Columbia, MO USA; 2 Department of Anthropology, Baylor University, Waco, TX USA; 3 Department of Anthropology, University of Texas at San Antonio, San Antonio, TX USA; 4 School of Data Science, University of Texas at San Antonio, San Antonio, TX USA

**Keywords:** Ecology, Environmental social sciences

## Abstract

Isolated indigenous societies who actively avoid sustained peaceful contact with the outside world are critically endangered. Last year, “Tanaru”, the lone surviving man of his tribe for at least 35 years, died in Southwest Amazonia, marking the latest cultural extinction event in a long history of massacres, enslavement, and epidemics. Yet in the upper reaches of the Amazon Basin, dozens of resilient isolated tribes still manage to survive. Remote sensing is a reliable method of monitoring the population dynamics of uncontacted populations by quantifying the area cleared for gardens and villages, along with the fire detections associated with the burning of those clearings. Remote sensing also provides a method to document the number of residential structures and village fissioning. Only with these longitudinal assessments can we better evaluate the current no-contact policies by the United Nations and governments, along with the prospects for the long-term survival of isolated tribes. While the world’s largest isolated indigenous metapopulation, Pano speakers in Acre, Brazil, appears to be thriving, other smaller isolated populations disconnected from metapopulations continue to be extremely vulnerable to external threats. Our applied anthropological conservation approach is to provide analyses of publicly available remote sensing datasets to help inform policies that enhance the survival and well-being of isolated cultural groups.

## Introduction

Colonization of Amazonia by non-indigenous populations has led to catastrophic demographic collapses of indigenous populations^1–3^. The irreversible threats from large-scale habitat loss via deforestation and conversion of land to agriculture and pasture potentially paint a bleak future for indigenous populations^4,5^, and these threats have intensified under recent political regimes who sought to undermine legal authority to reduce deforestation^6^. Despite the incessant external pressures from the outside world, some remote protected areas in the upper Amazon watershed support remnant indigenous societies referred to as uncontacted or isolated populations^7,8^. Between 30 and 50 isolated indigenous societies are estimated to remain in Amazonia, but most of their populations are small and fragmented^9,10^. The United Nations and most governments espouse no-contact policies for these isolated indigenous populations with the belief that they are safest if left to themselves^11^. If this “leave-them-alone” policy is successful, populations should demonstrate long-term population viability and growth. The long-term viability of such a strategy is doubtful, however, given a history of illegal incursions by resource-extraction outfits and the ever-present potential for disease epidemics^12^. One line of argument is that the history of disease, displacement, violence, and exploitation by outgroups after contact means that isolation and legal protections are the only means to ensure long-term survival for these groups^13^. Where protection of isolation is favored, there are numerous strategies to protect isolated communities^14^. Regardless, understanding the population dynamics and growth of these uncontacted groups and the risk of encroachment and incursion by resource extraction is vital for their long-term viability alongside further legal protection.

Unfortunately, despite being isolated from the outside world, these indigenous societies face uncertain prospects for long-term survival from pressures far beyond their control^14^. News about isolated indigenous societies is mostly tragic, from violent encounters with outsiders to illegal exploitation of their land and resources to cultural and linguistic extinction, as in the recent death of “Tanaru”^15^. The COVID-19 pandemic elevated the risk for indigenous groups who experienced outbreaks and elevated excess death rates^16–18^. The pandemic has also resulted in elevated resource conflicts for indigenous Amazonian communities, including areas of mining and resource theft^19^. Increased resource extraction in the Amazon is a salient threat to isolated indigenous groups as 97% of mining requests to the National Mining Agency in Brazil are in indigenous lands with isolated groups^20,21^. That some isolated societies have survived and maintained traditional lifeways is remarkable given that powerful external forces from logging, mining, poaching, narcotrafficking, and disease pose direct existential threats^20–25^.

Evidence that some isolated populations may be growing and thriving would offer renewed hope for their long-term survival and provide some support for no-contact policies. Direct investigation into isolated indigenous population dynamics is challenging both ethically and logistically. For example, it is impossible to collect census data, and overflights are unreliable for population estimates as people often actively avoid detection or are unobservable under living structures or the rainforest canopy. Hence remote monitoring is a useful method to study isolated indigenous demography and spatial ecology^26,27^. In contrast to overflights, remote sensing is safe and noninvasive. However, remote sensing approaches necessarily rely on indirect proxies of population size, such as the total cleared area of villages and gardens, the number of fire detections from burning associated with forest clearing, the number and size of living structures, and the occurrence or not of village fissioning^9^. Also crucially important is the need to remotely assess the spatial resource needs of indigenous societies in a region heavily impacted by deforestation and other threats. For example, using geospatial data to trace the effects of oil spills in the Ecuadorian Amazon highlights how oil spills impact hydraulic systems and threaten indigenous communities, including uncontacted groups^28^. Remote sensing can, therefore, be part of a larger geospatial toolkit to safely and remotely assess the outside threats to the health and livelihoods of isolated indigenous groups.

An important aspect of the long-term viability of populations is population substructure. In metapopulation ecology, a crucial component of demographic resilience is the “rescue effect” where spatially discrete reproductive subpopulations are linked through migration^29^ The vulnerability of local subpopulations to localized catastrophic events, disease outbreaks, or environmental and demographic fluctuations is mitigated through reproductive links with other subpopulations subject to their own set of vulnerabilities within the larger metapopulation. As a result, metapopulations, or reproductive connections amongst subpopulations, contribute to population viability^30,31^ as they display increased resilience over single populations by displacing localized risk. This metapopulation structure is likely important for isolated human groups in Amazonia. Local isolated groups are undoubtedly subject to many local vulnerabilities, enhanced by the catastrophic demographic collapse of larger-scale metapopulations over the last few centuries. Remnant, isolated populations are vulnerable to both demographic and environmental stochasticity due to their small sizes and the added risk that they are also likely no longer embedded within fully functioning metapopulations.

**Figure 1:**
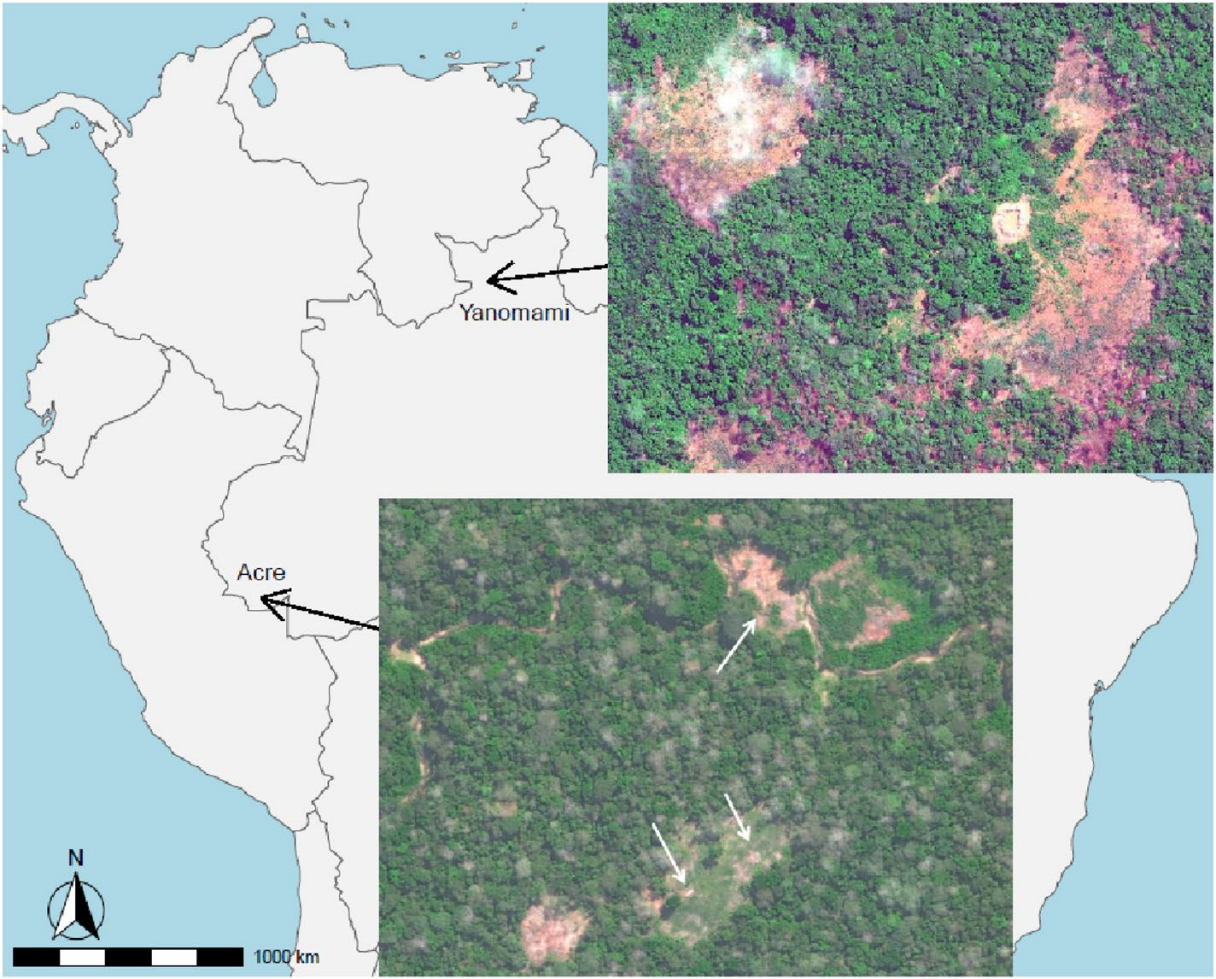
Top: Approximate locations for uncontacted Yanomami in their circular village (*shabono*), 55 m in diameter, on May 5, 2016. Bottom: example cluster of longhouses (white arrows), each about 20 m long, of uncontacted Pano speakers in Acre, Brazil, on August 6, 2015. High-resolution (50 cm per pixel) WorldView-03 imagery was purchased from Maxar Technologies, Inc.

Given the numerous threats to health and livelihoods of indigenous communities via encroachment, disease, and resource extraction, as well as intrinsic threats of population decline and collapse, remote assessments of isolated groups can provide information to influence policy on environmental protections as well as the ongoing debate over isolationism versus controlled contact. We join the chorus of others calling for additional study, including safe and remote monitoring of isolated indigenous communities, to better understand the vulnerabilities and threats these communities face^14,20,21^. The information yielded from these studies is necessary to safeguard the future of isolated indigenous communities. The violent and deadly history of past contact events implies that the collection of remote data is the only possibility to understand the threats to the health and well-being of these groups. Here we explore remote sensing methods to examine longitudinal change in land use of two isolated Amazonian groups (Fig. 1) to help assess population health and long-term viability.

## Results

### Uncontacted Pano speakers in Acre

**Figure 2:**
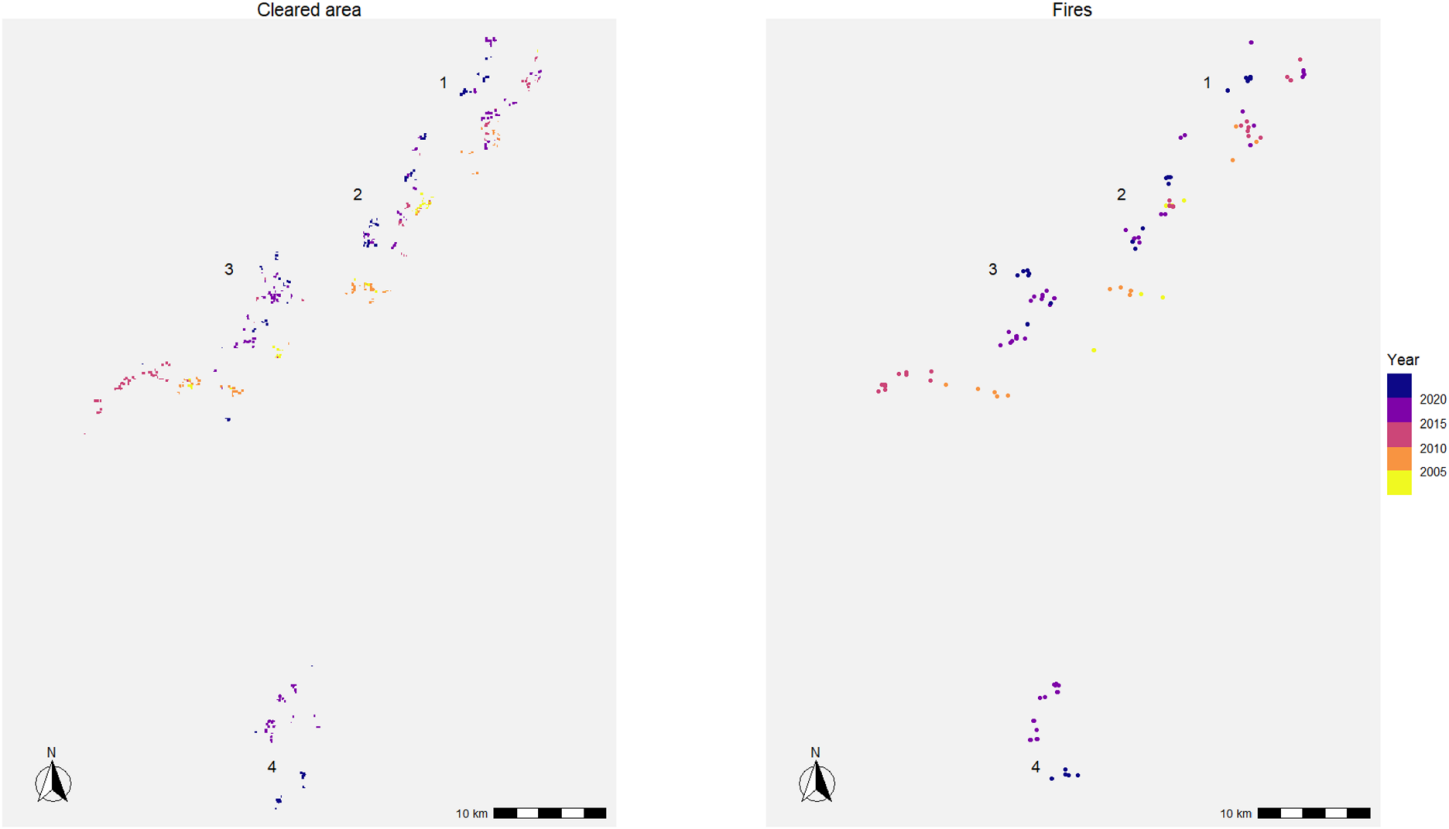
Similar ecological footprints of cleared area (left) and fires (right) made by uncontacted Pano speakers in Acre. Since 2000 they have spread in three directions and now live in at least four clusters of longhouses and gardens (labeled).

**Figure 3:**
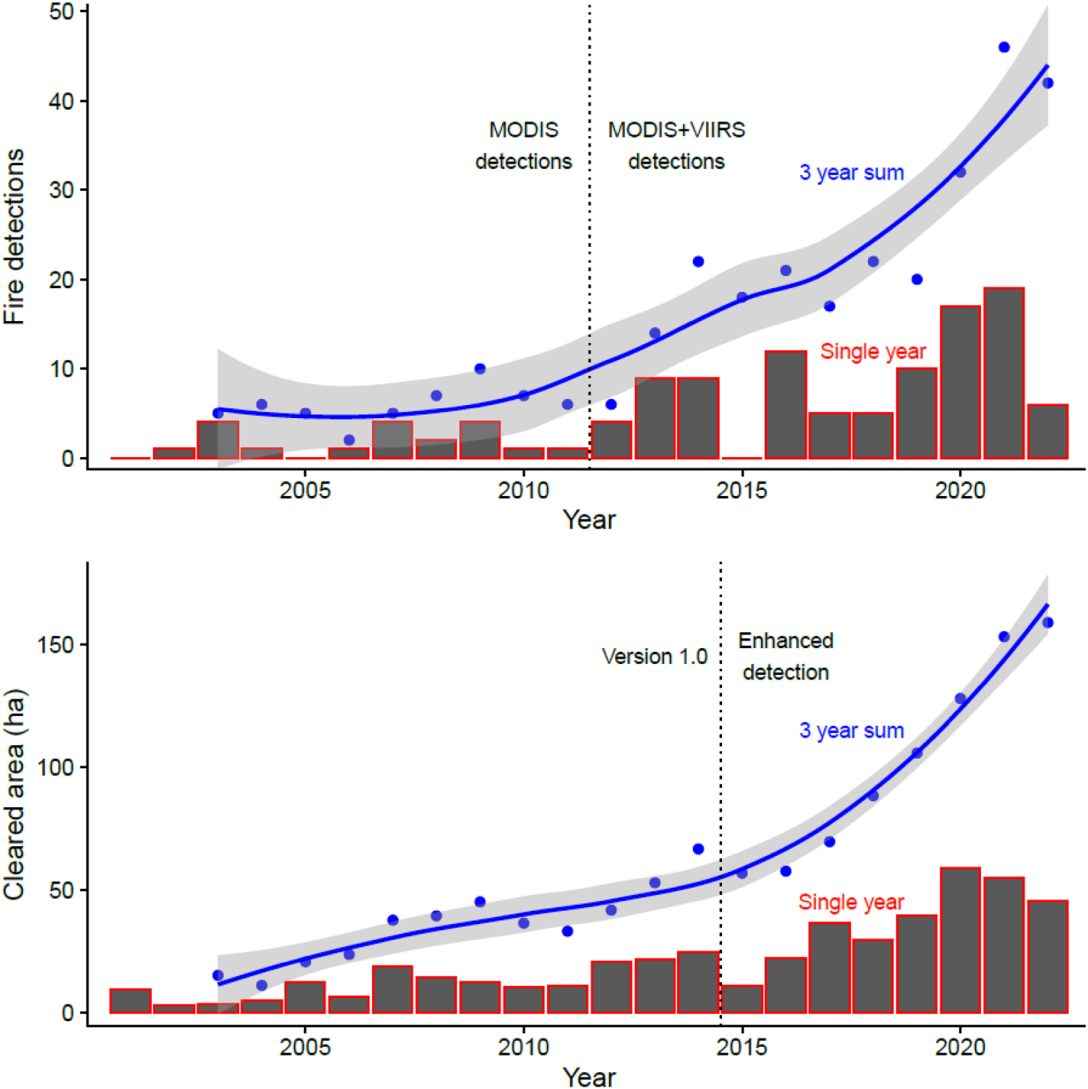
Fire detections (top) and cleared areas (bottom) have increased through time for uncontacted Pano speakers in Acre. Because gardens (mainly sweet manioc) are productive for around three years, a three-year running sum is one way to estimate total productive land. The three-year sums are fit with loess smooth curves. Technological and algorithmic improvements were made to satellite remote sensing that improved detection for fires after 2012 and for cleared areas after 2015. Fires are increasing at a rate of 14% per year since 2012, and cleared areas are increasing at a rate of 17% since 2015.

The world’s largest known isolated tribe, known locally by their watershed name Rio Humaitá, lived in multiple clusters of longhouses around the year 2000 and now live in at least four separate longhouse and garden clusters (Fig. 2). This metapopulation spread in three directions over the last few decades with a clear fissioning to the south in 2016. A total of 116 fire detections are attributed to the burning to clear their villages and gardens which now totals 460 ha. The annualized number of fire detections and cleared areas are strongly correlated (Pearson correlation coefficient = 0.83), and the rolling three-year sum of fires and cleared areas are even more strongly correlated (0.96), with regression slopes of 2.5 (0.37 S.E.) and 3.3 (0.24 S.E.) ha per fire, respectively. Importantly, the rate at which forest clearing and fire detections have increased through time is approximately exponential (Fig. 3), with three-year rolling sums increasing at a rate of about 14% per year (0.03 S.E.) for fires since 2012 and 17% (0.01 S.E.) for the cleared area since 2015. We use these cutoff years for better comparability to match the improved detection periods for more satellite sensors and improved algorithms.

There are other isolated tribes nearby. The Mashco Piro are nomadic hunter-gatherers and hence nearly impossible to track with satellite imagery, but a single horticultural village, likely Pano speakers, on the Peruvian side of the border shows a troubling remote sensing trajectory. From 2006 to 2015 this small village of Pano speakers cleared an average of only around 1.3 ha per year, indicating a tiny population (< 50 individuals), assuming horticulture is the main mode of subsistence. In 2016 and 2017 they cleared around 3 ha which would seem to be a positive trend. However, over the last five years there is no remote sensing signature either in forest clearing or fire detections that give any indication of their current location. Another example is the Txapanawa (Pano speakers) who made contact in 2014 in Acre after fleeing massacres in Peru. Similarly, their remote sensing footprint prior to contact was also small, only about one ha per year and no fire detections as they moved their only longhouse every few years. Hence the large uncontacted metapopulation of Rio Humaitá is exceptional given the size of their overall ecological footprint and their fast rate of growth.

### Isolated Yanomami in Roraima

**Figure 4:**
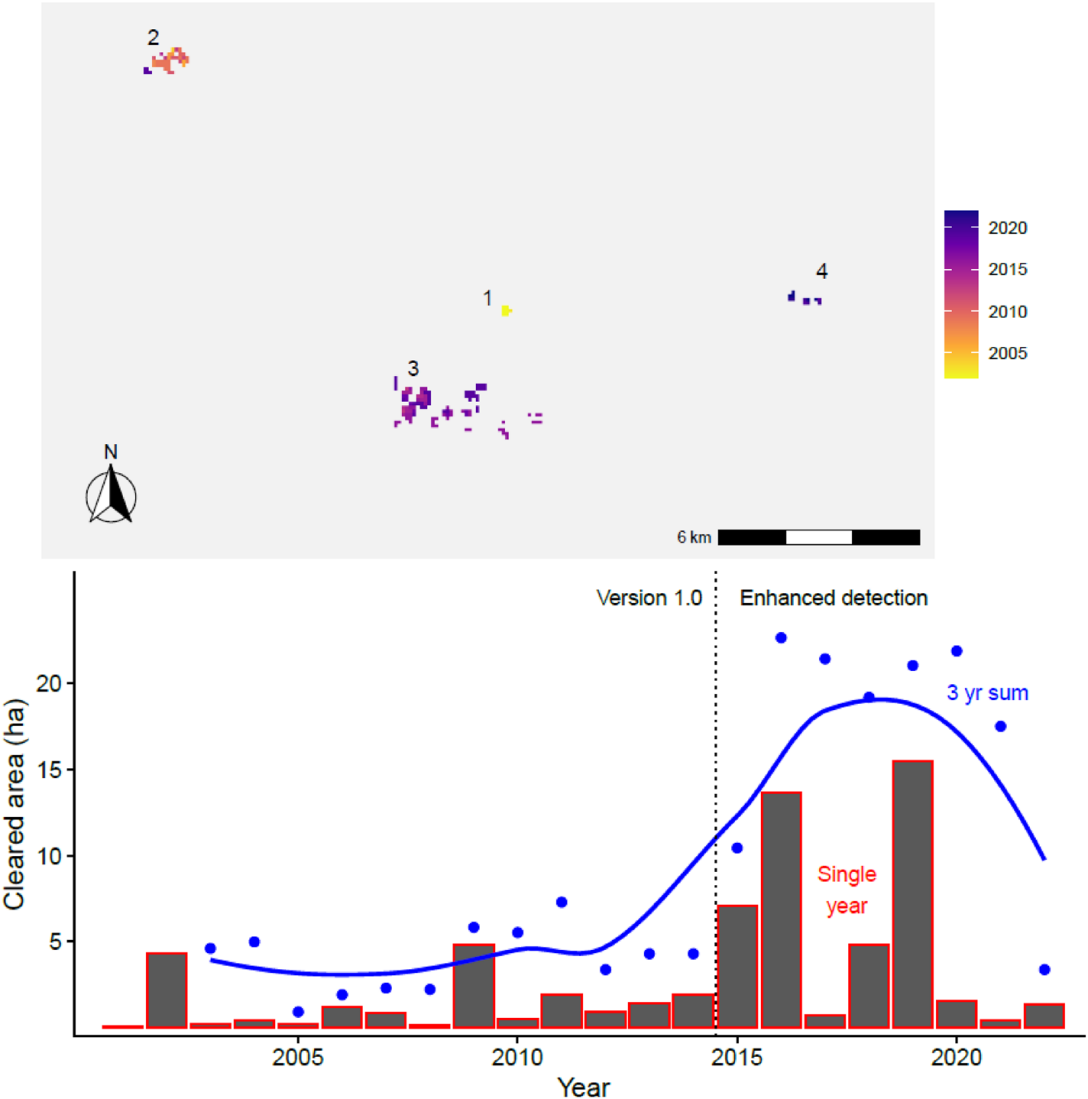
Uncontacted Yanomami have lived in four different locations in the last 23 years. Location three is notable for the size of its cleared area and 10 fire detections, while the most recent location four is notable because it has two *shabonos*.

The only remaining group of Yanomami to remain isolated from external contacts, known locally as the Moxihatëtëa, have moved three times over the last two decades. Their transition from location two to location three (Fig. 4) was well documented by overflights as it was originally feared that a catastrophe had occurred. Fortunately, they were found to have moved 13 km back towards their original location, were clearing large gardens, and the number of sections in their circular village, known as a *shabono*, had increased from 16 to 17. This third location is the only location to have fires detected 10 times in 2016 when much of the new village was cleared and in 2019 when most of the new gardens were cleared. In 2020 they moved again 12 km to the east and made two *shabonos* less than 1 km apart from each other with a shared garden in between. Together the two *shabonos* now house 23 sections. This is potentially good news and suggests a budding metapopulation. However, there are also troubling reports of violent encounters with nearby illegal gold miners (the closest mine is 15 km away) leading to Moxihatëtëa deaths^32^. Their current location has yet to show convincing evidence of growth, certainly not with the same exponential increase as seen in Acre. The overall cleared area made by these Yanomami total 64 ha since the year 2000. While remarkable, this is still only 14% of the total area cleared by the isolated metapopulation of Rio Humaitá.

## Discussion

We present some of the first positive evidence for sustained population growth in an uncontacted metapopulation using remote sensing imagery. The factors responsible for the success at Rio Humaitá merit further study and provide tentative empirical support for current no-contact policies. We now have evidence that isolated populations in certain situations have the capacity to grow demographically. Our results are a positive indicator for the long-term survival of currently isolated populations provided they are large enough and in relatively stable situations, such as the Rio Humaitá metapopulation. It is of course vital that they be allowed to maintain access to protected habitats that are large enough to support their subsistence needs and continued population growth.

We know post-contact Amazonian indigenous populations have been observed to grow at 3–4% per year on average^33^. The data we present here estimates recent growth of the extent of forest clearings and frequency of fire detections of isolated Rio Humaitá metapopulation to be 17% and 14% per year, respectively. This seems to be a surprisingly fast rate of growth. Of course, it is unclear how this activity rate in their ecological footprint maps onto demographic growth from reproduction and survival. Much of this growth may be related to increased access to steel tools that facilitate forest clearing. Significant immigration from neighboring isolated groups seems unlikely because there are only a few reports of other uncontacted groups in this area and they are all almost certainly quite small. Hence, sustained demographic increases, both actual and anticipated, are likely a good explanation of the results of the Rio Humaitá metapopulation. Populations rebounding from demographic or environmental shocks that include high mortality of young and old individuals are known to show high population growth rates because surviving adults reproduce quickly. Additionally, the stochastic effects of small population sizes can result in rapid growth rates, enhanced by positive density-dependence at small population sizes (i.e. Allee effects)^31,33,34^.

Isolation and hostility towards the outside world may serve as protection and may help improve chances for long-term viability by reducing risk of disease exposure. The COVID-19 pandemic affected nearby contacted indigenous populations (e.g. Refs.^17,35^), but we cannot know how uncontacted groups might have been affected by the pandemic. Uncontacted North Sentinelese in the Andaman Islands is one example of an indigenous population where isolation is arguably positive for long-term survival if the land and resources are sufficient to support a demographically viable population^36^.

In Acre, the intermittent and often hostile contacts between isolated tribes and indigenous and non-indigenous neighbors appear to increase over time. The straight-line distance between an isolated indigenous village and the closest contacted indigenous village is only 13 km. These intermittent contacts, especially during dry seasons when they make longer distance movements, are sometimes violent, involving shootings and thefts, with increased risk of disease transmission. However, neighboring contacted indigenous tribes are also known to leave gifts of machetes, axes, and other desirable items, and even plant gardens in hopes of discouraging violence and theft by their isolated neighbors. Interactions amongst the isolated tribes may also involve hostilities or avoidance^37^.

As mentioned above, an important aspect of the long-term viability of populations is not just access to habitat, resources, and exposure to disease, but population substructure. As local populations grow, they fission into spatially-discrete subpopulations which will have important additional positive impacts on demographic health and longer-term resilience. Given that metapopulations are expected to display increased long-term population resilience over single populations, it is reassuring that the Rio Humaitá have exhibited a metapopulation structure since the beginning of the study period. More cautiously, the potentially budding metapopulation of Yanomami is also reassuring. These metapopulation structures are important given the fact that local isolated groups are undoubtedly subject to many local vulnerabilities. Hence the lack of a metapopulation as seen in most other uncontacted tribes is troublesome as it does not bode well for long-term survival.

Remote monitoring is an important tool to help facilitate informed decisions and increase protection efforts of isolated indigenous groups. Limited efforts are being made to protect the rights and territories of uncontacted tribes and minimize outside forces' impact on their way of life. The future cultural survival of these tribes remains uncertain, and much will depend on the success of these efforts and the ability of the tribes themselves to adapt to changing circumstances. The no-contact policy that most governmental and non-governmental organizations promote for isolated indigenous populations is only logically defensible if their populations are indeed viable in the long-term. For the Rio Humaitá metapopulation, this does indeed appear to be the case, at least for now. The most important lesson is that long-term isolation may be viable in some cases provided isolated populations are large and growing and ideally interconnected in a metapopulation structure. Unfortunately, for most of the other smaller isolated indigenous populations separated from metapopulations, the encroaching external pressures raise a much more serious threat to their immediate survival. Our study demonstrates how publicly available data, methods, and computational techniques can be used to address issues of vital concern in the conservation of indigenous peoples. Using remote sensing and other geospatial tools, academics, conservationists, and policymakers can safely and ethically collect data to make informed decisions to help protect these communities from the numerous threats to their livelihoods.

## Methods

This paper focuses on the two largest isolated tribes in Amazonia. Isolated Pano speakers in Acre have grown to a population of 500–1000 people, while the isolated component of Yanomami is currently estimated at less than 100 people. Other known uncontacted populations (e.g. Carabayo in Colombia, Waorani in Ecuador, and Pano speakers in the Javarí Valley, Brazil and others in Peru) all appear smaller and have ephemeral forest clearing signatures with limited evidence for any growth and no fire detections^9^. The Global Forest Change (GFC) project provides longitudinal deforestation data at approximately 30 m per pixel resolution from Landsat sensors extending back to 2000^38^. For our purposes, GFC proved to be superior to other deforestation products for estimating the ecological footprint of uncontacted villages and gardens with an average underestimation of 11% compared to clearings measured with available high-resolution satellite images such as those in Fig. 1^9^. We used GFC data in *R* software 4.2.2 and the *terra* package to calculate the total area cleared by both the isolated Pano speakers in Acre and the isolated Yanomami in Roraima over the last 20 + years by digitizing boundaries around their known village and garden locations. We refer to this measure as cleared area, including villages, home gardens, and swidden fields. Technological and algorithmic improvements to satellite remote sensing improved detection for cleared areas after 2015^39^. Therefore, data are only strictly comparable either before or after that year. We include all clearings detected by GFC in the vicinity of known villages yielding a dataset comprised of 6,059 deforested pixels (460 ha) for uncontacted Pano speakers in Acre. For the Yanomami dataset, there are 828 deforested pixels totaling 64 ha.

The isolated Pano speakers demonstrate a temporal pattern to their ecological footprint of consistent growth in cleared areas of their villages and gardens, along with corresponding increases of forest burning that trigger fire detections from satellite data. The Yanomami show some marginal increases through time and evidence for a recently forming metapopulation. We estimated the total amount of arable horticultural land available to each group by summing areas cleared over the previous three years. Multi-year crop production time frames are characteristic of slash-and-burn horticulture for indigenous Amazonians such as those studied^40,41^. Pano speakers rely mainly on gardens of sweet manioc, and Yanomami gardens are mostly plantains. Both crops are productive for multiple years, so we use a three-year rolling sum to estimate total productive land use.

Finally, we identified burned forest clearings in the focal areas using remote sensing fire detection products from NASA derived from MODIS and VIIRS sensors^42,43^. MODIS is available starting in 2001 through the present, while higher resolution detection is available from VIIRS from 2012 onwards. MODIS and VIIRS detections consistently appear at the end of the dry season (mid to late March for the Yanomami and August–September in Acre). In the creation of villages and fields, forested areas are cut down, left to dry until the end of the dry season, and then burned. If these fires are sufficiently large, they trigger fire detections. Cleared area and fire detections give similar spatial patterning (Fig. 2), although the forest clearing data are more sensitive (i.e. higher resolution). We downloaded all available fire detection data for both focal areas up until the present. Data are only strictly comparable before or after 2012, so we focus our time trend analysis on the combined fire data after 2012. A three-year running sum of fire detections is used for Pano speakers. There are not enough fire detections for a Yanomami time series to be constructed as fires are only detected at their location three.

## Data Availability

The *R* script for our results is available at https://github.com/RobertSWalker/uncontacted. However, data are not made publicly available given the sensitive nature of disclosing the exact locations of uncontacted tribes.

## References

[1] Black FL (1975). Infectious diseases in primitive societies. Science.

[2] Hurtado M, Hill K, Kaplan H, Lancaster J (2001). The epidemiology of infectious diseases among South American Indians: A call for guidelines for ethical research. Curr. Anthropol..

[3] Walker RS, Sattenspiel L, Hill KR (2015). Mortality from contact-related epidemics among indigenous populations in Greater Amazonia. Sci. Rep..

[4] Le Tourneau FM (2015). The sustainability challenges of indigenous territories in Brazil’s Amazonia. Curr. Opin. Environ. Sustain..

[5] Ferrante L, Gomes M, Fearnside PM (2020). Amazonian indigenous peoples are threatened by Brazil’s Highway BR-319. Land Use Policy.

[6] Perez R (2021). Deforestation of the Brazilian Amazon under Jair Bolsonaro’s reign: A Growing ecological disaster and how it may be reduced. Univ. Miami Inter-Am. Law Rev..

[7] Castillo BH (2004). Indigenous Peoples in Isolation in the Peruvian Amazon: Their Struggle for Survival and Freedom.

[8] Ricardo FP, Gongora MF (2019). Cercos e Resistências: Povos Indígenas Isolados na Amazônia.

[9] Walker RS, Kesler DC, Hill KR (2016). Are isolated indigenous populations headed toward extinction?. PLoS One.

[10] Amorim FF (2016). Povos indígenas isolados no Brasil e a política indigenista desenvolvida para efetivação de seus direitos avanços, caminhos e ameaças. Rev. Bras. Linguíst. Antropol..

[11] Hosmanek AJ (2005). Indigenous homeland security: A proposed united nations draft declaration on the rights of indigenous peoples and the international law of first contact. Soc. Sci. Res. Netw..

[12] Walker RS, Hill KR (2015). Protecting isolated tribes. Science.

[13] Castillo, B. H. Respect for the self-determination and protection of the indigenous peoples in isolation. International Work Group for Indigenous Affairs (2021).

[14] Ortiz-Prado E, Cevallos-Sierra G, Vasconez E, Lister A, Pichilingue Ramos E (2021). Avoiding extinction: The importance of protecting isolated Indigenous tribes. AlterNative.

[15] The Last Member of an Uncontacted Tribe in Brazil Has Died | Smart News| Smithsonian Magazine. https://www.smithsonianmag.com/smart-news/last-member-uncontacted-tribe-brazil-died-180980671/.

[16] Cuéllar L (2022). Excess deaths reveal the true spatial, temporal and demographic impact of COVID-19 on mortality in Ecuador. Int. J. Epidemiol..

[17] Henriquez-Trujillo AR (2021). COVID-19 outbreaks among isolated Amazonian indigenous people, Ecuador. Bull. World Health Organ..

[18] Ramírez JD (2020). SARS-CoV-2 in the Amazon region: A harbinger of doom for Amerindians. PLoS Negl. Trop. Dis..

[19] Menton M, Milanez F, de Souza JMA, Cruz FSM (2021). The COVID-19 pandemic intensified resource conflicts and indigenous resistance in Brazil. World Dev..

[20] Villén-Pérez S, Moutinho P, Nóbrega CC, De Marco P (2020). Brazilian Amazon gold: Indigenous land rights under risk. Elem. Sci. Anthr..

[21] Villén-Pérez S, Anaya-Valenzuela L, Conrado da Cruz D, Fearnside PM (2022). Mining threatens isolated indigenous peoples in the Brazilian Amazon. Glob. Environ. Change.

[22] Zhouri A (2010). ‘Adverse forces’ in the Brazilian Amazon: Developmentalism versus environmentalism and indigenous rights. J. Environ. Dev..

[23] Shepard GH, Rummenhoeller K, Ohl-Schacherer J, Yu DW (2010). Trouble in paradise: Indigenous populations, anthropological policies, and biodiversity conservation in Manu National Park, Peru. J. Sustain. For..

[24] Salisbury DS, Fagan C (2013). Coca and conservation: Cultivation, eradication, and trafficking in the Amazon borderlands. GeoJournal.

[25] Yamada EM, Amorim FF (2016). Povos indígenas isolados: Autonomia e aplicação do direito de consulta. Rev. Bras. Linguíst. Antropol..

[26] Walker RS, Hamilton MJ (2014). Amazonian societies on the brink of extinction. Am. J. Hum. Biol..

[27] Walker RS, Hamilton MJ, Groth AA (2014). Remote sensing and conservation of isolated indigenous villages in Amazonia. R. Soc. Open Sci..

[28] Rivera-Parra JL, Vizcarra C, Mora K, Mayorga H, Dueñas JC (2020). Spatial distribution of oil spills in the north eastern Ecuadorian Amazon: A comprehensive review of possible threats. Biol. Conserv..

[29] Brown JH, Kodric-Brown A (1977). Turnover rates in insular biogeography: Effect of immigration on extinction. Ecology.

[30] Levins R (1969). Some demographic and genetic consequences of environmental heterogeneity for biological control. Bull. Entomol. Soc. Am..

[31] Hanski I, Gilpin M (1991). Metapopulation dynamics: Brief history and conceptual domain. Biol. J. Linn. Soc..

[32] Garimpo ameaça indígenas isolados em área Yanomami | Agência Brasil. https://agenciabrasil.ebc.com.br/direitos-humanos/noticia/2023-02/garimpo-ameaca-indigenas-isolados-em-area-yanomami.

[33] Hamilton MJ, Walker RS, Kesler DC (2014). Crash and rebound of indigenous populations in lowland South America. Sci. Rep..

[34] Hamilton M, Walker R (2018). A stochastic density-dependent model of long-term population dynamics in hunter-gatherer populations. Evol. Ecol. Res..

[35] Goha A (2021). Indigenous people and the COVID-19 pandemic: The tip of an iceberg of social and economic inequities. J. Epidemiol. Community Health.

[36] Sasikumar M (2019). The sentinelese of North Sentinel Island: A reappraisal of tribal scenario in an Andaman Island in the context of killing of an American preacher. J. Anthropol. Surv. India.

[37] Meirelles JC (2018). Terras compartilhadas por povos indígenas isolados e contatados: O Alto Rio Envira como estudo de caso. Tipití.

[38] Hansen MC (2013). High-resolution global maps of 21st-century forest cover change. Science.

[39] Forest Monitoring, Land Use & Deforestation Trends | Global Forest Watch. https://www.globalforestwatch.org/.

[40] Chagnon NA (1974). Studying the Yanomamo.

[41] Ross EB (1978). Food taboos, diet, and hunting strategy: The adaptation to animals in amazon cultural ecology. Curr. Anthropol..

[42] Justice CO (2002). The MODIS fire products. Remote Sens. Environ..

[43] Davies, D. et al. NASA’s fire information for resource management system (FIRMS): Near real-time global fire monitoring using data from MODIS and VIIRS. *NASA Technical Reports GSFC-E-DAA-TN73770* (2020).

